# A pilot randomized controlled trial of a tailored smoking cessation program for people living with HIV in the Washington, D.C. metropolitan area

**DOI:** 10.1186/s13104-020-05417-3

**Published:** 2021-01-06

**Authors:** Elexis C. Kierstead, Emily Harvey, Denisse Sanchez, Kimberly Horn, Lorien C. Abroms, Freya Spielberg, Cassandra A. Stanton, Charles Debnam, Amy M. Cohn, Tiffany Gray, Manya Magnus, Minal Patel, Raymond Niaura, Jessica L. Elf

**Affiliations:** 1 Schroeder Institute, Truth Initiative, 900 G St. NW, Washington, DC USA; 2 Department of Epidemiology, Milken Institute School of Public Health, The George Washington University, Washington, DC USA; 3 The Henry M. Jackson Foundation for the Advancement of Military Medicine, Bethesda, MD USA; 4 Carilion Fralin Biomedical Research Institute at VTC, Virginia Polytechnic Institute and State University, Blacksburg, VA USA; 5 Department of Population Health Sciences, Virginia-Maryland College of Veterinary Medicine, Virginia Polytechnic Institute and State University, Blacksburg, VA USA; 6 Department of Prevention and Community Health, Milken Institute School of Public Health, The George Washington University, Washington, USA; 7 Department of Population Health, Dell Medical School, University of Texas, Austin, TX USA; 8 Behavioral Health and Health Policy Practice, Westat, Rockville, MD USA; 9 Community Wellness Alliance, Washington, DC USA; 10 Department of Pediatrics, University of Oklahoma College of Medicine, Oklahoma City, OK USA; 11 Department of Community Health Administration, Department of Health, Washington, DC USA; 12 Department of Social and Behavioral Sciences, School of Global Public Health, New York University, New York City, NY USA; 13 Department of Environmental and Radiological Health Sciences, College of Veterinary Medicine & Biomedical Sciences, Fort Collins, CO USA

**Keywords:** HIV/AIDS, Tobacco, Cessation, Behavioral health, Minority health

## Abstract

**Objective:**

Morbidity and mortality from smoking-related diseases among people living with HIV (PLWH) in the U.S. surpasses that due to HIV itself. Conventional smoking cessation treatments have not demonstrated strong efficacy among PLWH. We conducted a pilot randomized controlled trial (RCT) to evaluate a tailored smoking cessation intervention based on the minority stress model. We compared standard of care counseling (SOC) to a tailored intervention (TI) including one face-to-face counseling session incorporating cognitive behavioral therapy to build resilience, and 30 days of 2-way text messaging.

**Results:**

The primary outcome was smoking cessation. Secondary outcomes included cigarettes per day (CPD), exhaled carbon monoxide (CO), and cessation self-efficacy. A total of 25 participants were enrolled (TI:11, SOC:14), and 2 were lost to follow-up. There were no significant differences in quit rates between study groups. However, there was a significantly greater decrease in CPD in the TI versus SOC (13.5 vs. 0.0, p-value:0.036). Additionally, self-efficacy increased in both groups (TI p-value:0.012, SOC p-value:0.049) and CO decreased in both groups (TI p-value: < 0.001, SOC p-value:0.049). This intervention shows promise to support smoking cessation among PLWH. A larger study is needed to fully evaluate the efficacy of this approach.

Clinical trial: Trial Registration: Retrospectively registered (10/20/2020) NCT04594109.

## Introduction

Cigarette smoking prevalence among U.S. adults is currently 14% [1]. However, for people living with HIV (PLWH), smoking rates are 2 to 3 times higher, ranging from 40 to 60% [2–6]. In addition to a high smoking prevalence, PLWH are also at increased risk of negative health effects from smoking as compared to their seronegative peers [3, 6–11]. As PLWH are living longer due to advances in treatment, the morbidity and mortality attributed to smoking-related diseases among PLWH in the U.S. currently outweighs that due to HIV infection [12]. Importantly, more than two out of three PLWH who smoke cigarettes are interested in quitting [4, 13] however, standard smoking cessation strategies have shown only moderate efficacy [14].

Washington, D.C. (D.C.) has one of the highest rates of HIV in the U.S. (1.8%) [15]. Additionally, among D.C. residents who are 50 or older, 3.2% are living with HIV [15]. The burden of disease falls most heavily on black men who have sex with men, as they make up the largest proportion of prevalent cases and newly diagnosed cases in D.C. [15]. According to data from a national sample, PLWH are at twice the risk of having an income at or below the poverty threshold [16]. Being a part of intersecting minority populations can lead to increased likelihood of mental health disorders and substance use, specifically tobacco [15, 17, 18]. A 2015 cross-sectional sampling of PLWH in D.C. found 40.6% identified as current smokers [19].

Prior research has described the ways in which stigma surrounding HIV has exacerbated smoking among PLWH [17], however limited research exists to combat smoking through specifically targeting these underlying determinants [14, 20]. We developed and evaluated a tailored smoking cessation intervention for PLWH in the D.C. metropolitan area that integrates aspects of minority stress theory [18] utilizing counseling, pharmacotherapy, and mobile health (mHealth) methodologies. An interim analysis of this work has been presented virtually at the Society for Behavioral Medicine’s annual conference in 2020 [21, 22].

## Main text

### Methods

We evaluated a newly developed tailored smoking cessation program for PLWH in D.C. via a pilot randomized controlled trial. This study was approved and overseen by the Chesapeake Institutional Review Board (IRB), now Advarra. All participants provided written informed consent prior to study enrollment.

A convenience sample of smokers was recruited via flyers in clinics and community centers serving PLWH, and word of mouth from September to December of 2017. Follow-up continued through February of 2018. Given the nature of the pilot study, sample size was determined based on feasibility and cost considerations. Participants were eligible if they were 1) 18 years or older, 2) living in the D.C. metro area, 3) self-reported PLWH, 4) currently smoking cigarettes daily, 5) willing to set a quit date within 7 days of the first meeting, 6) engaged in primary care, and 7) equipped with a mobile phone that could receive SMS text messages. At baseline, smoking status was confirmed by a carbon monoxide (CO) breath test where smoking was defined as a reading of greater than or equal to seven parts per million (ppm) using the coVita Smokerlyzer [23]. Participants were excluded if they were 1) using smokeless tobacco or electronic cigarettes at least once per day, 2) currently enrolled in a quit smoking program, 3) using nicotine replacement therapy (NRT), 4) currently in an alcohol treatment program, 5) pregnant, breastfeeding or planning to become pregnant, or 6) diagnosed with heart disease or high blood pressure that was not controlled by medication. They were also excluded if they had a heart attack in the last 2 weeks, serious underlying irregular heartbeat, serious or worsening chest pain, or active TMJ syndrome.

Once determined eligible and consented, participants were randomly assigned 1:1 to either the intervention or control arm. A computer-generated randomization sequence was used for individual randomization. Neither the participant nor study staff were blinded. Outcomes were determined at a follow-up visit one month later. The trial was completed at the conclusion of the funding period.

#### Study conditions

*Control Condition* Participants were provided a one-time standard of care (SOC) in-person cessation counseling session lasting approximately one hour and received a 30-day supply of nicotine replacement therapy (NRT) consisting of nicotine gum and patches. The SOC cessation counseling was adapted from the current clinical practice guidelines [24].

*Experimental Condition* Participants in the intervention arm were provided a one-time tailored cognitive-behavioral therapy (CBT) in-person cessation counseling intervention (TI) lasting approximately one hour, a 30-day supply of NRT, and a tailored bi-directional text messaging program delivering two messages per day for four weeks. Participants were asked to bring their mobile phones and were instructed by study staff how to initiatie the text message program during their baseline visit. Two participants in the TI group were not enrolled due to mobile-phone difficulties at baseline. The TI session was adapted from the clinical practice guidelines to include the elements of our conceptual framework rooted in the minority stress model [18, 24]. The intervention used a CBT approach to address issues of stress related to HIV stigma, minority status and socioeconomic condition. The conceptual framework supporting the TI theorizes that resilience-based coping has the potential to attenuate the negative effect of stigma on the relationship between stress and smoking. Positive coping strategies delivered to participants through the intervention are theorized to improve self-efficacy to cease smoking in the face of stress, adapting Teti et al.’s work on resilience [25–28].

#### Measures

At the baseline session, information was collected on sociodemographic characteristics, smoking patterns, past cessation attempts, and perceived access to quitting resources.

Our primary outcome of interest was self-reported smoking cessation at 30-days after baseline, verified by a CO reading of less than seven ppm. Secondary outcomes included changes in cigarettes per day (CPD), CO levels, and self-efficacy for cigarette abstinence [29].

Self-efficacy for cigarette abstinence was evaluated using a validated tool from Spek et al. wherein participants were asked to rate their confidence in not smoking given certain situations [29]. Response options included a five-point scale ranging from, “certainly not” to “certainly,” and the tool contained 6 items. Values 0–4 were assigned to the response options for each item and values were averaged to create a summary score.

#### Statistical analyses

Participant characteristics and demographic variables obtained at baseline were compared across treatment groups using Pearson’s Chi Squared or Fisher’s Exact tests, as appropriate, for categorical variables. Wilcoxon Mann–Whitney tests were used to evaluate differences between the study arms for continuous variables. Frequencies and percentages were reported for categorical variables while medians and inter-quartile ranges (IQRs) were reported for continuous variables. To assess the primary outcome of smoking cessation, cessation status at follow-up was compared across treatment groups and Fisher’s exact test was performed to assess differences between treatment arms.

An intent-to-treat (ITT) analysis was used wherein participants lost to follow-up (n = 2) were left in the dataset and were treated as if they did not quit and as if their continuous outcomes had not changed from baseline to follow-up. Scales for secondary outcomes were summarized by median value and change from baseline to follow-up. Change in scale from baseline to follow-up was evaluated across treatment groups using Wilcoxon Signed Rank Sum tests. All analyses were conducted using SAS 9.4 [30].

### Results

#### Participant characteristics

**Fig. 1:**
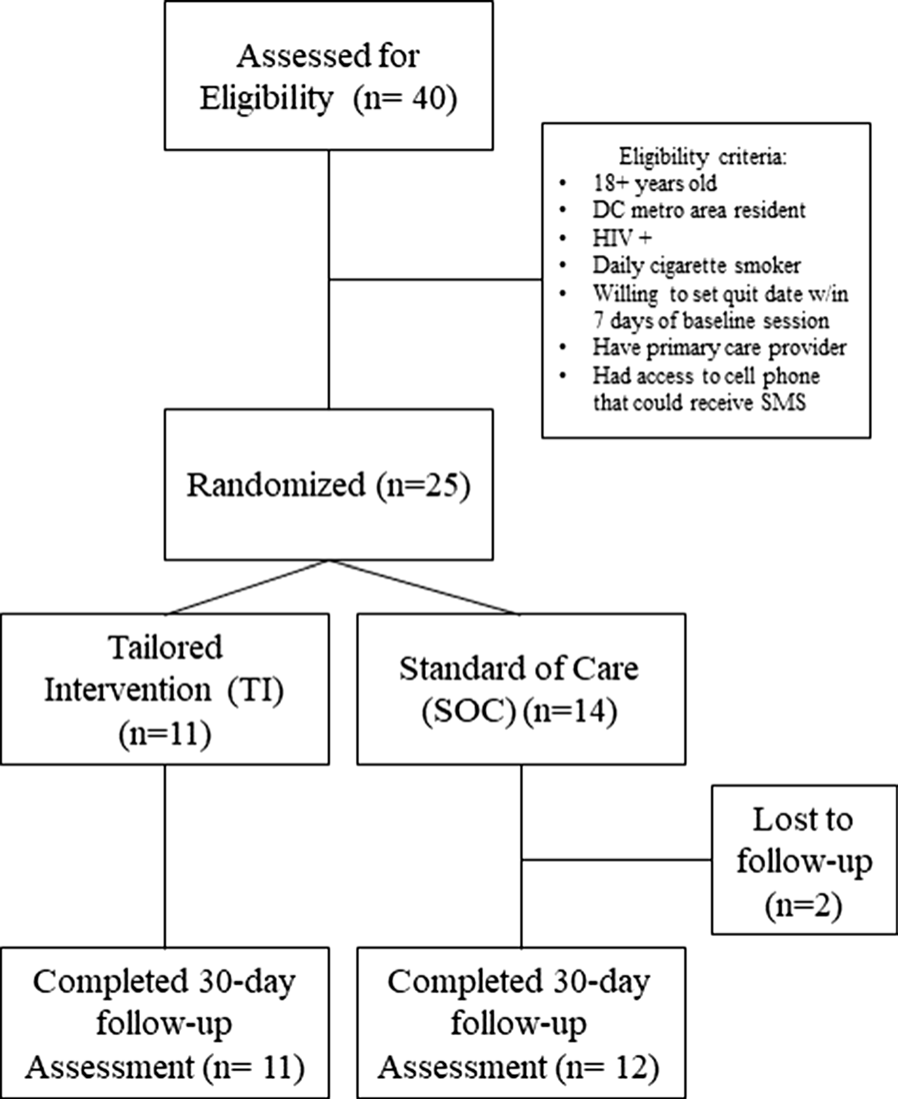
Participant flow through a randomized controlled trial of a tailored smoking cessation intervention for people living with HIV in the Washington, D.C. metropolitan area. Participants were assessed for eligibility based on screening criteria, randomized to a study arm, either a tailored intervention (TI) or standard of care (SOC), and asked to complete a 30-day follow-up assessment. Forty were assessed for eligibility, 25 were randomized (11 to TI and 14 to SOC) and 23 completed the 30-day follow-up assessment

Forty people were screened for the study and n = 25 were enrolled, with two participants lost to follow-up. Those screened and not enrolled either did not meet eligibility criteria or did not attend their scheduled session. Eleven were randomized to the TI (44%) and fourteen to the SOC (56%) (Fig. 1). The median age of participants was 54 (IQR: 48, 58). The majority were male (n = 18; 72%), Black or African American (n = 19; 76%), not Hispanic (n = 24; 96%), unemployed (n = 16; 64%), and making less than $20,000 a year (n = 19; 76%). Approximately half identified as a sexual minority (n = 13; 52%). The median age at which the participants were diagnosed with HIV was 32 (IQR: 25, 43) and the median amount of time the participants had lived with HIV was 19.5 years (IQR: 15, 28.5). There were no statistically significant differences between study arms (Table 1).

**Table 1:** Demographic characteristics of participants enrolled in a randomized controlled trial of a tailored smoking cessation intervention for people with HIV in the Washington, D.C. metro area, by treatment group (n = 25)

	Total (n = 25)	Control (n = 14)	Intervention (n = 11)	P-value*
	Median (IQR)	Median (IQR)	Median (IQR)	
Age	54 (48, 58)	51 (47, 57)	58 (51, 60)	0.0715
Cigarettes per day at baseline	10 (3, 20)	9 (1, 15)	15 (6, 20)	0.1224
Age first smoke regularly	18 (16, 20)	18.5 (16, 23)	16 (15, 19)	0.2173
Years smoking regularly	37 (27, 41)	32 (23, 38)	38 (35, 44)	0.0507
Age at HIV diagnosis	32 (25, 43)	31 (25, 37)	32 (24, 44)	0.4025
Years living with HIV	19.5 (15, 28.5)	19 (15, 27)	20 (13, 28)	0.9085

	n (%)	n (%)	n (%)	
Gender identity				0.8037
Male	18 (72)	11 (79)	7 (64)	
Female	5 (20)	2 (14)	3 (27)	
Other/Transgender	2 (8)	1 (7)	1 (9)	
Race				0.1804
Not Black or African American	6 (24)	5 (36)	1 (9)	
*White*	*1 (4)*	*1 (7)*	*0 (0)*	
*American Indian or Alaska Native*	*1 (4)*	*1 (7)*	*0 (0)*	
*More than one race*	*4 (16)*	*3 (21)*	*1 (7)*	
Black or African American	19 (76)	9 (64)	10 (91)	
Ethnicity				0.4400
Not of Hispanic, Latino or Spanish origin	24 (96)	14 (100)	10 (91)	
Of Hispanic, Latino or Spanish origin	1 (4)	0 (0)	1 (9)	
Sexual Identity				1.000
Straight/heterosexual	12 (48)	7 (50)	5 (45)	
Sexual minority	13 (52)	7 (50)	6 (55)	
*Gay or lesbian/homosexual*	*9 (36)*	*6 (43)*	*3 (27)*	
*Bisexual*	*3 (12)*	*0 (0)*	*3 (27)*	
*Something else*	*1 (4)*	*1 (7)*	*0 (0)*	
Education				1.000
Some College or more	15 (60)	8 (57)	7 (64)	
High School/GED/Vocational or Less	10 (40)	6 (43)	4 (34)	
Employment				0.2077
Employed	9 (36)	7 (50)	2 (18)	
Not Employed	16 (64)	7 (50)	9 (82)	
Financial needs				0.4347
Meeting needs and more	11 (44)	5 (36)	6 (55)	
Just/Not Meeting needs	14 (56)	9 (64)	5 (45)	
Household income per year				0.6036
≥ $20,000	4 (16)	3 (23)	1 (10)	
< $20,000	19 (76)	10 (77)	9 (90)	
Prefer not to say	2 (8)	1 (7)	1 (9)	
Menthol cigarette smoking				0.2300
Non-menthol	3 (12)	3 (21)	0 (0)	
Menthol	21 (84)	11 (79)	10 (91)	
No usual brand	1 (4)	0 (0)	1 (9)	
Used of NRT during the study period	21 (84)	11 (79)	10 (91)	0.6043
Tried to quit smoking in the past 12 months	13 (52)	8 (57)	5 (45)	0.6951
Were advised to quit smoking in the past 12 months by a doctor or healthcare provider	7 (28)	4(29)	3 (27)	1.000

Race and sexual minority categories were condensed for statistical testing due to small sample size, however the frequency and percentages of the condensed categories are shown in italics

*p-value comparing treatment arms

At baseline, participants smoked a median of 10 (IQR: 3, 20) cigarettes per day. In the prior 12 months, approximately half of participants had tried to quit smoking (n = 13; 52%). The median age at which participants had first smoked regularly was 18 (IQR: 16, 20). Most reported that they had not been advised to quit by a healthcare provider in the past 12 months (n = 18; 72%). Thirty percent did not know if quit smoking services are available at their place of HIV care and 17% reported that these services are not available (Table 1).

Nine participants (82%) interacted with the text messaging program beyond initiating the program at the baseline session. Participants received a median of 71.5 text messages (IQR: 61, 75) and sent a median of 8 text messages (IQR: 3, 10). Participants sent a minimum of 2 and a maximum of 22 text messages throughout the study. Most participants self-reportedly used the NRT provided (n = 21; 84%).

#### Quitting outcomes

In total, eight participants quit smoking by follow-up (32%); this included five participants in the SOC group (35.7%) and three participants in the TI group (27.3%). All participants who self-reported that they had quit had CO levels below 7 ppm, verifying their status. Among those who did not quit, those in the TI group experienced significantly greater decreases in CPD than the SOC group (13.5 vs. 0.0, p = 0.0358). There were significant differences between baseline and follow-up CO in both treatment groups (TI p = 0.0488, SOC p = 0.0005). Additionally, both the groups experienced significant increases in self-efficacy from baseline to follow-up (TI p = 0.0117, SOC p = 0.0488) (Table 2).

**Table 2:** Primary and secondary outcomes of participants enrolled in a randomized controlled trial of a tailored smoking cessation intervention for people with HIV in the Washington, D.C. metro area, from baseline to 30-day follow-up, by treatment group (n = 25)

	Control				Intervention				P-value^d^
	Baseline	Follow-up			Baseline	Follow-up			
	n (%)	n (%)			n (%)	n (%)			
Cessation	–	5.0 (35.7)			–	3.0 (27.3)			1.0000

	Median (IQR)	Median (IQR)	Median (IQR) Change	p-value^c^	Median (IQR)	Median (IQR)	Median (IQR) Change	p-value^c^	
Cigarettes per day^a^	9.0 (1.0, 15.0)	0.5 (0.0, 3.0)	− 0.5 (-15.0,0.0)	0.0586	15.0 (6.0, 20.0)	3.0 (0.0, 10.0)	− 10.0 (− 19.0, − 3.0)	0.0020	0.1652
Cigarettes per day among those not quit^b^	3.0 (1.0, 10.0)	3.0 (1.0, 5.0)	0.0 (0.0,1.0)	0.8125	20.0 (9.0, 25.0)	4.0 (2.0, 11.0)	− 13.5 (− 19.5, − 2.5)	0.0156	0.0358
Carbon Monoxide (ppm)	14.0 (10.0, 24.0)	7.0 (4.0,14.0)	− 5.0 (− 9.0,4.0)	0.0005	19.5 (11.0, 23.5)	12.5 (11.5, 20.0)	− 4.5 (− 9.0, 1.0)	0.0488	0.4172
Self Efficacy (Scale: 0-4)	1.8 (1.0, 2.5)	2.5 (1.5, 3.0)	0.3 (0.0, 1.0)	0.0488	1.0 (0.5, 2.5)	2.0 (2.0, 4.0)	1.0 (− 0.5, 2.0)	0.0117	0.4989

^a^Cigarettes per day in the total sample. All those who quit have a CPD of zero. (n = 25)

^b^Cigarettes per day in those who did not quit. All those who quit excluded. (n = 17)

^c^Significant differences tested from baseline to follow-up

^d^Significant differences tested between study groups

### Discussion

Our pilot project of a tailored smoking cessation program for PLWH in the Washington, D.C. metropolitan area showed promising results. The majority of participants in the intervention group were actively engaged in the text messaging program. Both the TI and SOC groups had 30-day cessation rates at around 30%. The intervention arm, however, saw significantly greater reductions in CPD among those who did not quit than the control arm. Both arms demonstrated significant decreases in CO and significant increases in self-efficacy to abstain from smoking from baseline to follow-up. These data were analyzed ITT. Although there was variation in significance and magnitude, the overall trends did not change when the sample was analyzed “as-treated.”

Although participants receiving the intervention in this small study were not more likely to quit than those who received SOC, it is important to note that 32% of the total sample (n = 8) did successfully quit smoking at one month. According to the CDC, less than 1 in 10 smokers have quit successfully in the past year despite approximately half of all smokers reportedly making a quit attempt [31]. Additionally, in a comparable cessation intervention for PLWH that also had a 30-day follow-up among a larger sample of 95 smokers living with HIV, Vidrine et al. evaluated a cell phone delivered counseling intervention and found that those in the study quit smoking at a rate of 8% in the control group and 21% in the intervention group; lower quit rates than what can be seen in this study. Although this sample did not see a significant difference between the intervention and control groups, the rates at which participants quit smoking after only 30-days was on par with previous research [32].

Additionally, although the control group received a “standard of care” cessation counseling treatment, it is unlikely this is truly the standard of what PLWH are experiencing in their regular clinical care as few participants reported having been advised to quit by their healthcare providers in the prior year. Although PLWH are consistently engaged with the healthcare system, providers may be missing opportunities to intervene and may not be providing standard of care counseling at the level necessary to create change.

These data suggest that this tailored intervention could be a promising strategy for smoking cessation among PLWH. Though it was not shown to be superior to a standard of care intervention in this small study for complete cessation, the intervention did significantly decrease number of cigarettes smoked, and positively impacted changes in self-efficacy, a key activating component of the conceptual model. Larger studies providing greater exposure to the counseling program are needed to fully evaluate the efficacy of this intervention.

### Limitations

- The sample was small, reducing the ability to account for possible cofounders via adjustment.
- Short follow-up time.
- Secondary outcomes were self-reported, not accounting for some biases.
- Due to enrollment errors, two of eleven participants in the treatment group did not receive the full text messaging program.

## Data Availability

This data is housed at Truth Initiative and is not publicly available.

## Abbreviations

PLWH: People living with HIV
RCT: Randomized controlled trial
D.C: Washington D.C
SOC: Standard of care
CBT: Cognitive behavioral therapy
TI: Tailored intervention
NRT: Nicotine replacement therapy
CO: Carbon monoxide
